# From chemistry to fruit flies: An unpredictable series of fortunate conversations

**DOI:** 10.1371/journal.ppat.1007077

**Published:** 2018-08-09

**Authors:** Sara Cherry

**Affiliations:** Department of Microbiology, University of Pennsylvania, Philadelphia, Pennsylvania, United States of America; The Fox Chase Cancer Center, UNITED STATES

Scientific discovery is wondrous and challenging. Learning new things and finding new ways to answer fundamental questions has driven me both intellectually and personally. I have also tried to make unique contributions: If someone else is doing the experiment, why should I do it? Moreover, I have been strongly shaped by my personal interactions and training environments. These experiences have provided me the confidence and expertise to answer unasked questions and to study diverse aspects of science, using ever-changing techniques and systems that are quite outside the box.

I began my scientific career in high school, where I worked over the summers exploring amoeba feeding habits and cocks-comb proteoglycan structures, each experience opening up new areas for me to explore. When I was thinking about college, I decided on the University of California–Berkeley in the College of Chemistry, as this path was completely devoted to science endeavors. There, I took an organic chemistry class with Peter Schultz that was transformative; he made chemistry come alive. I was so enamored by the class that I asked him if I could work in his lab. This conversation began my years in his laboratory, during which I became immersed in the science and in the laboratory environment. I worked as much as I could, collaborating with graduate students and postdocs to develop new backbones for peptide-based drugs, and I loved it. I became more independent through my time there, and I knew that this was what I wanted to do. Half of the lab at the time was developing antibodies to perform new tasks including chemistry, and I became interested in how antibodies were made. This led me to audit the Immunology Core class in the biology department since there was no room for outside classes in the College of Chemistry. This class was a turning point for me. It was taught by Astar Winoto, who, though I had not realized it at the time, had trained with David Baltimore. The class opened up the world of immunology to me, and many of the topics covered were ones that were heavily influenced by David’s research. This led me to contact David directly, leading to a Saturday morning meeting at the Rockefeller before I had applied to graduate school, and it was amazing. With his guidance, I applied to the biology department at the Massachusetts Institute of Technology, where he was relocating. The next year I showed up at his laboratory to discuss joining his laboratory focusing on the outstanding questions in antibody development and the intricate mechanisms behind allelic exclusion. We decided that this was a great place for me to start.

Going from chemistry to biology was a difficult transition, opening my eyes to completely new facets of science and new approaches. The lab environment was fantastic; it was like being in a mini-institute where everyone was excited and driven to new discoveries across diverse aspects of immunology. It was also hard core; everyone was at the top of their game and had a critical eye. I responded well to this intensity; I loved being in the lab learning, experimenting, and being challenged daily. The intellectual freedom was exciting and was exactly what I was looking for. Within the lab, there were a number of unbelievably impressive scientists working on HIV, and of course, David had made fundamental discoveries in virology. This began my fascination with viral infections. When I began looking for postdoctoral positions, I knew that I wanted to study viral infections, but I wanted to do it from a genetic perspective since some of the most exciting and novel findings are those that come from unbiased screens. However, at that time, there was a dearth of mammalian genetic systems amenable to systematic screening, so I began my quest for a new approach.

I wanted to screen for new genes that control viral infections and to do so using a system that would likely have parallels in humans. This led me to flies and to Norbert Perrimon, who has pioneered many new techniques and approaches using *Drosophila* and who is completely open to new ideas. When I pitched the use of fly genetics to study viral infections, he was all in. Learning to work with flies was exhilarating, and the power of genetics seemed limitless. Moreover, my proficiency in mammalian cell culture was a bonus at the time, as we began to set up high-throughput cell-based screening using the newly discovered RNAi system in fly cells. This led me to perform the first genome-wide RNAi screen for host factors involved in viral infections.

When I started my own lab at the University of Pennsylvania (UPenn) in 2006, I wanted to combine my expertise in diverse systems to fully explore the mechanisms by which viruses can establish infection in and across diverse hosts. And this drew me to the study of viruses that are transmitted from arthropods to humans. To me, these are the most amazing pathogens on earth. With only approximately 11 kb of coding information, these viruses can infect and replicate in divergent hosts while evading their immune systems. This has led my laboratory to explore the conserved mechanisms by which these viral pathogens can subvert and hijack host pathways. I have continued to use genetic systems to explore virus–host interactions, and more recently, this has led my laboratory to explore the interface between the microbiota and viral infections, performing genetic screens in bacteria and screening metabolites for their roles in antiviral defense.

I feel that my varied background and my passion for discovery will continue to serve me well as we explore new facets of microbiology and immunology. And my love of screening has led me to start a Core at UPenn, which has led me to help the scientific community to apply this approach to diverse aspects of science but also led me to translational research, and we have begun screening acute leukemia patients for sensitivities to approved therapeutics to personalize therapies. Working with clinicians to ultimately treat patients is incredibly rewarding. This is an exciting twist to my scientific career, and I am not sure where my explorations will lead next, but I am looking forward to it.

**Image 1:**
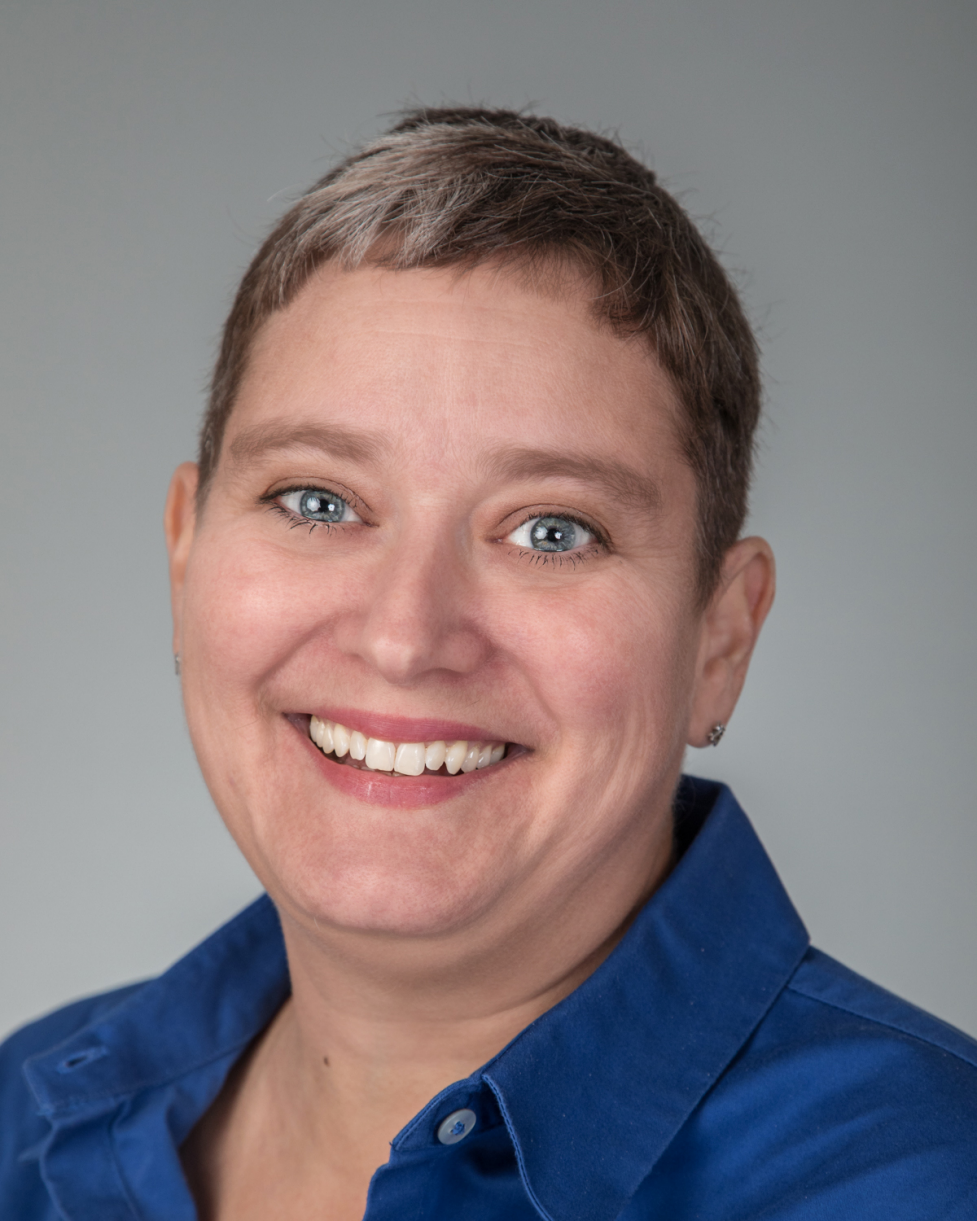
Sara Cherry.

